# Nanosurgery and Bioengineered Regenerative Protocols for the Treatment of Hip Osteoarthritis: A Double-Blind Randomized Controlled Trial as an Alternative to Surgical Hip Replacement

**DOI:** 10.3390/biomedicines13040987

**Published:** 2025-04-17

**Authors:** Cezary Wasilczyk, Bartosz Wasilczyk

**Affiliations:** Medical Department, Wasilczyk Medical Clinic, ul. Kosiarzy 37/80, 02-953 Warszawa, Poland; bwasilczyk@gmail.com

**Keywords:** hip, nanosurgery and bioengineering treatment (NSBT), hip osteoarthritis

## Abstract

**Introduction:** Hip osteoarthritis (HOA) significantly affects mobility and quality of life, with total hip arthroplasty (THA) being a common treatment. However, complications and increasing revision rates highlight the need for alternative approaches. This study evaluates the efficacy of ultrasound-guided nanosurgery and bioengineering treatment (NSBT) compared to non-standardized platelet-rich plasma (PRP) treatment for patients with symptomatic HOA. **Methods:** A double-blind, randomized trial included 38 patients referred for THA, divided into two groups. The study group received NSBT with modified PRP enriched with somatotropin and *Strophanthus kombe*, while the control group received PRP and hyaluronic acid injections without a standardized protocol. Treatments were guided by ultrasound, and outcomes were assessed using the Visual Analog Scale (VAS), Western Ontario and McMaster Universities Arthritis Index (WOMAC), Harris Hip Score (HHS), and range of motion (RoM) evaluations over 12 months. **Results:** The study group showed significant improvements in all clinical outcomes, including reductions in VAS scores from 7.8 to 0.2 (*p* < 0.0001) and WOMAC scores from 76.2 to 10.5 (*p* < 0.0001). The HHS improved from 56.4 to 93.0, and RoM showed substantial gains in flexion, external rotation, and internal rotation (all *p* < 0.001). The control group demonstrated less pronounced improvements. **Conclusions:** NSBT offers a safe and effective alternative for managing HOA, significantly reducing pain and improving joint function while potentially delaying or avoiding the need for THA. Further long-term studies are warranted to confirm these findings.

## 1. Introduction

Hip osteoarthritis (HOA) is one of the most common musculoskeletal disorders, affecting millions of people worldwide. Symptomatic hip osteoarthritis (SHOA) is characterized by pain, stiffness, and a limited range of motion in addition to degenerative changes seen on imaging studies. SHOA is associated with a reduced quality of life and limitations in daily living activities. In addition, it is a significant risk factor for disability, defined as the need for assistance when walking on a flat surface or climbing the stairs [1].

Total hip arthroplasty (THA) has become increasingly popular in recent decades and is currently one of the most common surgical procedures worldwide. The annual number of patients undergoing THA exceeds 1 million in the United States alone [2,3]. Despite advances in prosthetic technology as well as in surgical devices and techniques, hip replacement is still associated with the risk of an unfavorable outcome [4].

In recent years, there has been a dramatic increase in the number of patients referred for hip replacement. Moreover, there is a global trend for quick surgical referrals, with patients being scheduled for hip surgery already at the early stages of osteoarthritis. As a result, the mean age of patients undergoing THA has been decreasing. While the available literature usually reports satisfactory outcomes for THA, there is a sizable proportion of patients who do not benefit from the procedure. Recently, there have been an increasing number of referrals for revision THAs [2,3,4,5], and this constitutes a major challenge in the treatment of patients referred for the primary hip replacement procedure. Total hip replacement is not only a humanistic challenge but also a significant economic burden [5,6,7,8,9,10,11]. The tendency for the increasing number of referrals for THA and for the decreasing age of patients at surgery is paralleled by a trend to preserve the hip joint for as long as possible using arthroscopy and orthobiologic treatments. The aim of this approach is to postpone or avoid THA, while preserving optimal hip function in patients with SHOA. SHOA was reported to affect 27 million people in the United States, about 10% of men and 13% of women older than 60 years, and about 3% of the population older than 30 years [12,13]. The hip joint is the second largest joint in the human body, following the knee, and is commonly affected by OA. Currently, it is estimated that more than 1 million Americans undergo primary THA every year [14]. It is estimated that the increase in the volume of primary THA procedures was 34% in 2020 and 75% in 2025. This is projected to reach 129% by 2030 and 284% by 2040 [14]. There is an ongoing discussion about the clinical efficacy of THA, including patient satisfaction and health status outcomes. Based on the literature, the lack of satisfaction with treatment outcomes is reported in 7% to 25% of patients [15,16,17,18,19,20,21,22,23,24,25,26,27,28,29,30,31,32,33]. Moreover, THA was reported to be associated with a higher risk of hospitalization due to cardiovascular events and central nervous system diseases in the 9-year follow-up as compared with the control group, as well as with a higher risk of death in the long-term follow-up [34,35,36,37,38].

Considering the higher risk of complications and the annual increase in the rates of revision THAs, as well as the higher morbidity and mortality of patients after THA in long-term studies, there is a need to discuss alternative methods of treatment for symptomatic HOA. There is growing interest in orthobiologic injections for treating HOA. Although the number of studies and their methodologies are still limited, the available evidence indicates that these treatments are safe and generally yield promising results. Notably, the success of the treatment tends to be inversely proportional to the severity of HOA [39].

In this study, we assessed the effectiveness of a modified PRP treatment that incorporates growth hormones, specifically somatotropin and *Strophanthus kombe*, in precise doses. Somatotropin promotes anabolic activity in tissues, while *Strophanthus kombe*, a glycoside, inhibits the sodium–potassium pump, increasing intracellular sodium and calcium levels and enhancing cellular turgor. Glycosides have also been shown to influence the sympathetic–parasympathetic nervous system, boosting the sensitivity of cellular receptors, including baroreceptors, following injury [40,41]. The increase in cellular turgor and anabolic activity directly stimulates cellular memory. When combined with strategically selected injection sites, this approach initiates the regeneration of the injured hip.

The aim of the presented study is to assess the treatment efficacy of modified PRP using human cell memory (RP-hCM) intake in patients referred for primary THA, including patients undergoing ultrasound-guided nanosurgical and bioengineering treatment (NSBT), and of non-standardized orthobiologic treatment with percutaneous (PRP) delivery into the hip joint under ultrasound guidance.

The secondary aim is to develop standards for NSBT procedures and to systematize the methodology of ultrasound-guided NSBT of the hip joint in patients with symptomatic HOA.

## 2. Materials and Methods

### 2.1. Study Design

This double-blind randomized clinical trial (registration number: ISRCTN15642019) was carried out at the Wasilczyk Medical Clinic between 2022 and 2023. All participants signed written informed consent forms and were insured in accordance with European Union regulations.

All patients were previously referred for primary THA. The exclusion criteria were as follows: cancer, pregnancy, breastfeeding, hip injury in the 12 weeks before enrollment, active inflammation associated with rheumatic disease, and lumbar radiculopathy symptoms.

The NSBT of the hip was planned based on clinical examination and additional tests including the following:

1. Ultrasound assessment of hip morphology, and radiologic imaging with MRI where necessary.
2. Assessment of the joint range of motion before and after treatment (flexion, internal rotation in 90 degrees flexion, and external rotation in degrees flexion and abduction).
3. Clinical and ultrasound assessment to identify local pathological mechanisms underlying symptomatic HOA.
4. Pain assessment and evaluation of physical function in the activities of daily living during and after treatment, using the Visual Analog Scale (VAS), Western Ontario and McMaster Universities Arthritis Index (WOMAC), and the Harris Hip Score (HHS).
5. Assessment of structural changes using imaging studies.

In all patients, the same algorithm for the clinical examination of the hip joint was used:

1. Exclusion/confirmation of intracapsular and extracapsular inflammation based on ultrasound imaging.
2. Clinical and ultrasound assessment of the intracapsular, extracapsular, anterior, anteromedial, lateral, and posterolateral aspect of the hip.

Patients were randomized either to a group treated with a standardized NSBT treatment with RP-hCM intake or to a group treated with a non-standardized method using PRP and hyaluronic acid injected under ultrasound guidance and without a systematized treatment plan for the delivery of orthobiologics. A simple randomization model was used, with sequentially numbered, opaque, and sealed envelopes to conceal the allocation. Patients were blinded to which treatment group they were assigned and were unblinded at a 6-week follow-up visit. Data collectors and assessors were also blinded. An independent examiner was blinded to the nanosurgical and injection side and study group.

The sample size was determined based on treatment outcomes observed in pilot studies. To achieve a statistical power of 0.9, a minimum of 16 participants per group was required, assuming the effectiveness of NSBT was 60% in the study group and 25% in the control group, with a high standard deviation of 30%. To account for potential dropouts during the screening process, the study planned to recruit a total of 40 patients.

### 2.2. Intervention

The study compared two treatment approaches. The first group received a non-standardized method involving ultrasound-guided injections of PRP and hyaluronic acid, without a structured protocol for administering orthobiologic therapies.

In contrast, the second group underwent a standardized NSBT protocol, which included RP-hCM containing somatotropin and *Strophanthus kombe*, alongside hyaluronic acid and collagen. The RP-hCM was prepared aseptically by combining all components. PRP was obtained by centrifuging 6–10 mL of the patient’s blood at 1800 rpm for 8 min.

All treatments were administered percutaneously under local anesthesia and ultrasound guidance. Depending on individual clinical responses, patients in both groups could receive two or three treatment cycles, with injections spaced 2–4 weeks apart. MRI follow-ups were conducted nine months after treatment, while outcomes were evaluated at 12 months post-treatment. These outcomes included clinical assessments using the VAS, WOMAC, HSS, and range of motion (RoM) test.

### 2.3. Methodology of Nanosurgical and Bioengineering Procedures (NSBT)

**Figure 1:**
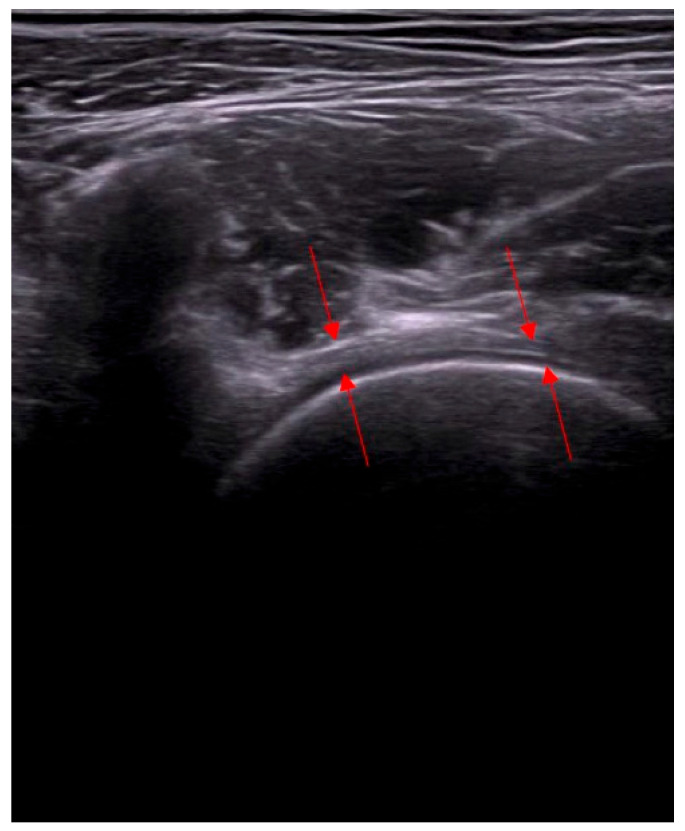
Ultrasound cross-section examination of the anterior compartment of the hip joint. Capsulitis adhesive of the front joint capsular complex—significant bolding of the joint capsule. Arrows indicate thickening of the capsular complex.

**Figure 2:**
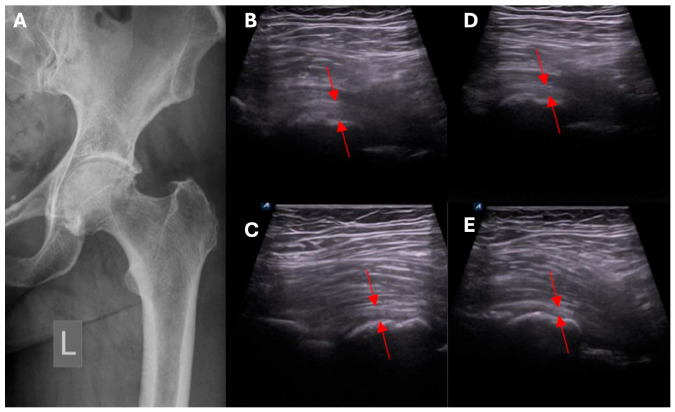
(**A**) Radiological image of the hip joint. (**B**–**E**) Ultrasound examination of the hip joint; red arrows indicate gradual narrowing of the front joint capsule over time following the administration of NSBT. L—left side.

The NSBT approach uses the elements of classic surgical operations, but the procedure is done percutaneously and under ultrasound guidance. With NSBT, it is possible to target and release the core points in the diseased parts of the hip joint. Techniques such as ultrasound-guided dry needling or injection were reported, but they have not been well described and have been used for the local delivery of a drug or biological material. The NSBT methods in the treatment of the hip joint include capsulotomy (Figure 1), and release of the proximal hamstring tendons, gluteal muscles, and planar adhesions of the fascia. So far, there have been no studies reporting ultrasound-guided hip capsulotomy (Figure 2), or NSBT release of gluteal muscles. Restriction of the range of the motion is not a contraindication to apply NSBT. The immediate outcomes of the procedure are satisfactory, with an improvement in the hip range of motion and function. For NSBT, an individualized plan for the separate compartments of the hip joint is developed depending on the local state of bone and soft tissue, considering RP-hCM and visco- or collagen supplementation.

The capsulotomy was performed in the area of the capsular complex thickening exceeding 1 mm. The technique involved microfenestration and adhesion dissection using a needle with a diameter of 0.6–0.8 mm. The procedure was conducted under ultrasound guidance, allowing for precise localization and assessment of pathological changes.

Due to the variability in hip joint anatomy, the procedure was performed at a depth ranging from 3 cm to as much as 7–8 cm in subcutaneous tissues. In real-time, the angle of capsular complex fenestration typically ranged between 30–45 degrees.

To ensure procedural reproducibility, the following standardization criteria were applied:

1. Assessment of capsular adhesion thickness—ultrasonographic evaluation was performed with the probe positioned transversely, allowing for precise visualization of adhesions. The most common location of adhesions was in the anterolateral complex, with less frequent occurrences in the medial complex.
2. Number and distribution of surgical portals—capsulotomy was performed using at least two portals, and in some cases, three or four, depending on the adhesion locations. Standardly, two access points were located in the anterolateral compartment—medially and laterally to the iliopsoas tendon.
3. Access to the medial capsular complex—in selected cases, the procedure was performed via a medial approach relative to the neurovascular bundle, which is a limitation of arthroscopic methods.
4. Anterior capsulotomy location—the procedure could include a capsulotomy at the equator of the femoral head in the transverse ultrasound view. Optional additional approaches included a medial approach relative to the neurovascular bundle.

The capsulotomy procedure was supported by biological agents that, in addition to NSBT effects, facilitated the lysis of capsular adhesions. The procedure was performed, on average, 2–3 times. During the procedure, the ultrasound probe was positioned transversely to the hip joint, enabling real-time assessment and adjustment of the instrument penetration depth. The optimal scanning depth was 5–8 cm.

### 2.4. Statistical Analysis

Numerical variables were reported by calculating means and standard deviations. Due to the lack of normality of the distribution (tested using the Shapiro–Wilk test), non-parametric tests were used: the Mann–Whitney U test, and the Wilcoxon test for paired comparisons. The threshold for statistical significance was set at α = 0.05. To account for the risk of a Type I error due to multiple between-group comparisons, Bonferroni correction was applied for all pairwise comparisons. For each set of related comparisons, the significance threshold was adjusted by dividing the standard alpha level (0.05) by the number of comparisons.

Statistical analysis was performed using GraphPad Prism version 10.0.0 (GraphPad Software, Boston, MA, USA, www.graphpad.com).

## 3. Results

The study involved 38 patients with SHOA, evenly distributed between the study and control groups. The baseline characteristics of the study and control groups were comparable. The mean age of participants in the study group was 66.4 ± 14.2 years, while in the control group it was 65.8 ± 16.7 years. Both groups had a similar sex distribution. The mean body mass index (BMI) was slightly lower in the study group (24.1 ± 2.2) compared to the control group (27.5 ± 4.1). Regarding disease grade, none of the participants were classified as grade I, while 15.8% in the study group and 5.3% in the control group were classified as grade II. There was more grade IV OA in the control group compared to the study group, 47.3% vs. 21.0% (Table 1).

**Figure 3:**
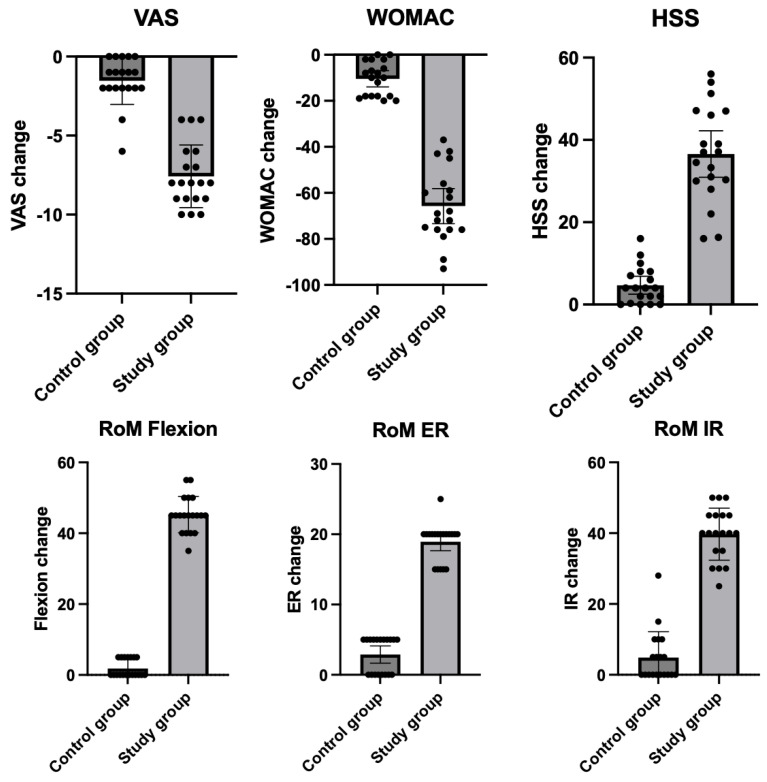
Mean changes in clinical scores for both the treatment and control groups 12 months after the procedure. Whiskers indicate the 95% confidence intervals, and dots represent individual patients. The differences in all parameters were statistically significant (*p* < 0.001). Abbreviations: ER—external rotation; HHS—Harris Hip Score; IR—internal rotation; RoM—range of motion; WOMAC—Western Ontario and McMaster Universities Arthritis Index; VAS—Visual Analog Scale.

The clinical outcomes of the study demonstrated significant improvements in the study group compared to the control group. In terms of pain reduction, the VAS decreased from 7.8 (95% CI: 6.9–8.6) at baseline to 0 (95% CI: 0.01–0.4) post-treatment in the study group (*p* < 0.0001), while the control group showed a more modest reduction from 8.8 (95% CI: 8.3–9.3) to 7.3 (95% CI: 6.3–8.2) (*p* = 0.0003). The WOMAC improved significantly in the study group from 76.2 (95% CI: 71.1–81.3) to 10.5 (95% CI: 5.1–15.9) (*p* < 0.0001), whereas changes in the control group were less pronounced. Similarly, the HSS score in the study group improved from 56.4 to 93.0 (*p* < 0.0001), compared to smaller improvements in the control group. The range of motion (RoM) also showed significant enhancements in the study group, with flexion increasing from 94.0° (95% CI: 91.6–96.3) to 139.2° (95% CI: 136.6–141.8) (*p* < 0.001), while the control group improved from 92.6° to 95.3° (*p* = 0.24). External rotation (ER) and internal rotation (IR) also demonstrated remarkable gains in the study group, with ER improving from 9.7° to 28.4° and IR increasing from 21.6° to 61.3° (both *p* < 0.0001), whereas the control group showed only minor changes. These findings underscore the superior clinical outcomes achieved in the study group (Table 2 and Figure 3).

As part of the sensitivity analysis, patients with grade IV OA were excluded from the dataset. At baseline, the control group (n = 10) showed slightly worse scores in VAS, WOMAC, and HSS compared to the study group (n = 14). Comparative analysis between the intervention and control groups revealed statistically significant differences in VAS scores post-intervention, as well as in the magnitude of change (*p* < 0.001 for all comparisons). Within-group analyses also demonstrated significant improvement after treatment in both cohorts. Consistent findings were observed for other clinical and functional parameters. The results are summarized in Supplementary Table S1.

**Figure 4:**
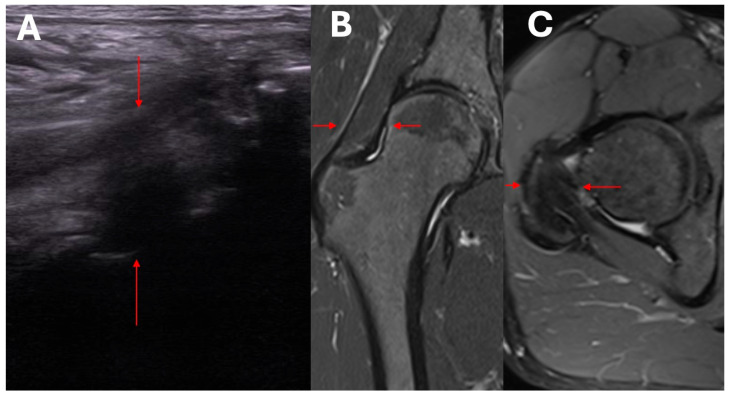
Lateral compartment of the hip joint with an assessment of the rotator muscles, demonstrating degenerative changes, adhesions, and edema-related alterations. (**A**) Ultrasound examination; and (**B**,**C**) MRI. The red arrows indicate the same tissue compartment in the same patient. In the ultrasound assessment, greater resolution of the area is observed.

Figure 4 presents the lateral compartment of the hip joint with an assessment of the rotator muscles, demonstrating degenerative changes, adhesions, and edema-related alterations. The significantly higher resolution and the ability to evaluate individual compartments are notable advantages of ultrasound examination.

### Safety

No serious adverse events were observed in either group. Despite the anatomical proximity of the neurovascular bundle, no cases of deep vein thrombosis were reported.

- Transient joint stiffness and inflammatory response occurred in some cases:
  ⚬ in one patient in the study group;
  ⚬ in two patients in the control group.
    All resolved spontaneously within 48 h.
- Mild pain at the injection site, lasting until the following day, was reported by three patients in each group.

Although a temporary reduction in range of motion was expected due to the relatively extensive nature of the procedure, no such limitation was observed in either group.

## 4. Discussion

There is an ongoing discussion about orthobiologics—their efficacy and indications for use—in orthopedics, including for the treatment of symptomatic HOA. In the past 20 years, there has been a dramatic increase globally in the number of patients referred for primary THA, and the age of these patients is clearly decreasing.

In our study, we used an innovative approach to non-invasive hip treatment by looking at the entire hip region based on a detailed clinical assessment and imaging tests. The treatment plan covered clinical and structural deficits in the different hip regions separately for the intracapsular and extracapsular compartments, gluteal tendinopathy, hamstring tendinopathy, and fascia structure. In our study, both the control and study groups showed improvement in their VAS, WOMAC, HSS, and range of motion parameters. However, the study group achieved significantly better results.

The effect of *Strophanthus kombe* and somatotropin on the treatment of HOA remains speculative, and further studies are needed to elucidate the molecular and cellular mechanisms underlying this potential therapeutic action. The regenerative effects observed in the NSBT group may be partially attributed to the biological activity of somatotropin and *Strophanthus kombe*, key components of the RP-hCM formulation. Somatotropin (growth hormone) plays a role in musculoskeletal tissue repair by stimulating protein synthesis, enhancing cellular hydration, and promoting cartilage and bone regeneration through the JAK2/STAT5 signaling pathways. *Strophanthus kombe*, a cardiac glycoside, modulates cellular turgor by inhibiting Na^+^/K^+^-ATPase, leading to increased intracellular sodium and calcium levels. This ionic shift activates downstream signaling cascades (e.g., PKC) which support cytoskeletal remodeling and extracellular matrix stabilization. Together, these mechanisms may help restore cellular homeostasis and enhance tissue resilience in osteoarthritic joints [42,43,44,45].

*Strophanthus kombe* has been shown to positively influence cell turgor by inhibiting Na^+^/K^+^-ATPase activity. This inhibition disrupts the intracellular ion balance, increasing osmotic pressure and thereby enhancing cell hydration and function. Basically, inhibition of Na^+^/K^+^-ATPase leads to an increase in intracellular sodium levels. This, in turn, disrupts the electrochemical gradient, resulting in an elevation of intracellular calcium via Na^+^/Ca^2+^ exchange mechanisms. Elevated intracellular calcium plays a crucial role in activating signaling cascades that promote cell survival and anabolic activity in musculoskeletal tissues [42]. In particular, increased calcium levels stimulate protein kinase C (PKC) and downstream effectors involved in cytoskeletal organization and extracellular matrix remodeling [43]. These molecular changes may facilitate the restoration of cellular homeostasis in osteoarthritic tissue, improving the biomechanical properties of affected joints. Similarly, somatotropin, commonly referred to as growth hormone (GH), plays a pivotal role in improving cell turgor by promoting cellular hydration and raising intracellular osmotic pressure. These effects are achieved through its anabolic functions, including the stimulation of protein synthesis and the regulation of ion transport. GH independently increases the size of differentiated myotubes without altering the size, proliferation, or differentiation of myoblast precursors. It facilitates the fusion of myoblasts with developing myotubes, leading to an increased number of myonuclei. This fusion process relies on NFATc2, as GH-induced hypertrophy is absent in NFATc2-deficient myotubes. Additionally, GH operates independently of insulin-like growth factor 1 (IGF-1) by utilizing distinct signaling mechanisms, as evidenced by the lack of IGF-1 regulation in myotubes, the absence of secreted mediators, and the additive effects observed when GH and IGF-1 are combined. Also, GH exerts anabolic effects on musculoskeletal tissues by enhancing protein synthesis, increasing extracellular matrix production, and modulating cellular hydration. GH stimulates the activation of the JAK2/STAT5 signaling pathway, which plays a fundamental role in regulating gene transcription associated with cartilage and bone regeneration [44,45]. These characteristics suggest a potential role of GH in muscle cell fusion and hypertrophy. The combined properties of *Strophanthus kombe* and somatotropin may hold promise in regenerative applications, particularly in supporting cellular recovery and structural maintenance. While speculative, this biological rationale when integrated with the NSBT method and a structured neurosurgical approach could help explain the favorable clinical outcomes observed in our study [42,43,44,45].

NSBT techniques have not been described before, although there have been some reports on dry needling or nanosurgical techniques. The nanosurgical technique for hip treatment has been systematized and standardized. The NSBT technique reflects surgical procedures but uses ultrasound guidance and core point release in the individual anatomical structures within each hip compartment. The NSBT treatment of the hip joint includes capsulotomy and the release of the proximal hamstring tendons, gluteal muscles, and fascia adhesions. To our knowledge, no previous studies described percutaneous capsulotomy of the hip. The immediate outcome of the NSBT procedure is improvement in hip mobility. To date, we have not observed any adverse events. The NSBT technique, being a nano-invasive approach, significantly reduces the risks typically associated with such procedures; however, the possibility of adverse effects cannot be entirely excluded. Following capsulotomy, transient inflammatory or edematous changes may occasionally occur, potentially leading to temporary pain-related restriction of joint mobility in the early postoperative period. Nevertheless, no such events have been observed in our cohort so far. It should also be emphasized that ultrasound-guided capsulotomy is a highly safe procedure, particularly considering the proximity of the neurovascular bundle. Real-time imaging allows for precise visualization and navigation of instruments, thereby minimizing the risk of iatrogenic injury.

The specifics of the treatment plan, including the number of procedures, their sequence, and time between the individual procedures, should be guided by local factors. The individual hip compartments should be assessed by ultrasonography. An individualized approach to the treatment of the patient with HOA should be adopted.

Despite improvements in prosthetic material and design as well as advances in surgical tools and methods, complication rates after primary THA remain the same. Patients referred for revision hip replacement after primary THA constitute a significant medical, humanistic, and economic challenge [4,5,6,7,8,9,10,11]. Global analyses show that the percentage of patients requiring revision THA remains high and continues to grow significantly [46,47,48,49,50,51,52,53,54]. According to some investigators, unfavorable outcome rates after primary THA are maintained at the level of 7%, but this is in contrast to other reports, in which the rate of patients referred for revision THA was 12% to 25% at 5 to 7 years [2,9,14,55,56]. These patients are also at additional risk of local and systemic complications after surgery, such as hip dislocation after primary THA, infections, periprosthetic fractures, and early and late vascular complications. The rate of prosthetic hip dislocation ranges from 0.3% to 10% (mean annual rate, 3–4%) [8,11,57,58,59,60,61,62,63,64,65,66].

Research shows that the highest risk of local and systemic complications is observed in patients referred for primary THA between the age of 50 and 75 years. Moreover, these patients are most likely to require revision hip replacement, with 12% to 35% of cases referred for the procedure within 5 to 8 years after primary THA [58,59,60,63,66,67,68]. At 9 years, patients who underwent THA had a higher risk of worse general health status and hospitalization due to cardiovascular and central nervous system events than matched controls who did not undergo surgical treatment. According to data from research, the risk of revision THA is significantly lower in patients older than 75 years.

The patients from this study are being followed up through phone calls and in-person visits when required. Some participants from both the control and study groups have follow-up periods extending up to two years. We plan to publish these results once the follow-up period increases further. Transient anterior hip pain lasting 2–3 days was noted in three patients. Apart from this, there were no associated complications in the study group, and the effects persist to this day. In the control group, despite initial improvement, after 12 months, there was a gradual worsening back to the pre-treatment clinical hip state.

In our study, we observed an almost complete resolution of pain in the NSBT group (VAS reduction from 7.8 to 0.2), which exceeds the effect sizes typically reported in trials involving patients with chronic HO. While this outcome may appear unusually favorable, it may be attributable to the comprehensive and anatomically targeted nature of our intervention. To our knowledge, no previous studies have addressed hip joint treatment with such detailed attention to specific anatomical compartments. Our protocol involved targeted management of (1) the gluteal muscles, whose dysfunction significantly limits internal rotation; (2) the external rotator muscles and lateral capsular complex at the femoral neck level, which restrict external rotation; (3) the adductor compartment, which impairs abduction; and (4) the capsular complex, where release procedures likely provided joint decompression. Meta-analyses of other orthobiologic treatment methods report lower reductions in VAS scores. In the case of mesenchymal stem cells (MSCs), a pooled analysis of 13 studies (742 patients) showed a mean difference in 12-month VAS scores of −1.4 (*p* = 0.002), which is notably less than the reduction observed in our study [69]. Similarly, the effectiveness of intra-articular PRP appears more limited. An analysis of five studies including 234 patients reported a mean VAS reduction of only −1.6 points at 12 months (*p* < 0.05), which is substantially lower than the outcomes achieved with the NSBT protocol in our study [70]. The results of the sensitivity analysis, performed after excluding patients with grade IV osteoarthritis, further support the effectiveness of the intervention. Although the control group demonstrated slightly worse baseline values in several functional parameters (VAS, WOMAC, and HSS), the magnitude of improvement observed in the study group was substantially greater across all key outcomes. Notably, the post-treatment scores for pain, function, and range of motion were significantly better in the study group, indicating a more robust response to the intervention.

Our findings support the growing body of evidence indicating the potential of orthobiologic therapies in managing hip osteoarthritis. In a recent meta-analysis including 18 randomized controlled trials and 1174 patients, Tian et al. [69] reported that MSC therapy resulted in a mean improvement in WOMAC scores of −15.94 points at 12 months (n = 454; 95% CI: −23.79 to −8.10; *p* < 0.0001). While these results support the functional benefits of MSC-based treatments, the magnitude of improvement remains modest compared to the outcomes achieved with the NSBT protocol in our study.

A recent systematic review and meta-analysis by Lim et al. [70], which included eight studies and 331 patients treated with intra-articular PRP for hip osteoarthritis, found that although PRP significantly improved pain outcomes, the improvement in functional scores (WOMAC, HOOS-ADL, and HHS) was limited. A statistically significant functional benefit was observed only at the early follow-up (1–2 months), while no significant improvement was maintained at later time points. These findings contrast with the more sustained improvements in function observed in our NSBT-treated cohort.

The efficacy of regenerative medicine, including the use of orthobiologics for the hip, is debatable. Xie et al. [11] reported that despite considerable healthcare resources for the treatment of HOA in the United States, the quality-of-life outcomes remain unsatisfactory. A considerable number of patients after primary THA will experience prosthesis loosening. Between 1 January 2009 and 31 January 2013 a total of 258,000 revision procedures were conducted in the United States [11], and this number is projected to double in the next decades.

In a narrative review, McInnis et al. [71] claimed that there is currently insufficient evidence to support the use of orthobiologics in the treatment of hip disease. However, the authors underlined the importance of understanding hip disease according to specific hip regions. They were also the first to classify hip disease by hip region.

Villanova-Lopez et al. [72] compared the outcomes of treatment with PRP vs. hyaluronic acid and reported a high efficacy of orthobiologics for hip disease. At the 12-month follow-up, a significant improvement was observed in hip function, pain relief, and the use of analgesia in patients who received PRP, as based on the HHS score among other assessment tools. However, the research included a follow-up of 12 months. According to the global data, such a timeframe post-PRP treatment typically sees a regression in treatment outcomes [72].

Sanchez et al. [73] also confirmed the high efficacy and safety of ultrasound-guided PRP injections in patients with severe hip osteoarthritis. In their prospective study, 40 patients received three intra-articular PRP injections at weekly intervals. Significant improvements in VAS, WOMAC, and Harris Hip Score pain subscales were observed at both 7 weeks and 6 months. Notably, 57.5% of patients experienced a clinically meaningful pain reduction of ≥30%, and 40% were classified as excellent responders with sustained improvements in both pain and disability. The reported side effects were minimal and limited to a transient sensation of heaviness at the injection site.

Dallari et al. [74] conducted a randomized clinical trial to compare the efficacy of ultrasound-guided transdermal injection of PRP, PRP + hyaluronic acid, and hyaluronic acid alone for the treatment of HOA. The outcomes were evaluated with the HHS, WOMAC, and VAS scores. At the 12-month follow-up, there was a significant improvement in pain and hip function in all patients, with the best outcomes reported for patients who received PRP injections.

Battaglia et al. [75] reported a higher efficacy of ultrasound-guided intra-articular injections of PRP compared with hyaluronic acid in patients with HOA. The highest efficacy of treatment was noted at 1 and 3 months in both groups, as assessed by VAS and HHS scores. However, between 6 and 12 months, worse outcomes in both groups were noted.

Fiz et al. [76] conducted a study in patients with HOA grade 3 according to the Tönnis classification to determine the effect of orthobiologics on the quality of life and prevention of or delay in primary THA. Ultrasound-guided intra-articular and intraosseous infiltration of PRP was performed in the operating room. Intraosseous infiltration was applied into the acetabulum and the femoral head using a fluoroscope under venous anesthesia. The authors reported satisfactory clinical outcomes at 6 months.

Dallaudiere et al. [77] investigated 408 patients with tendinopathy (including the adductor longus tendon). The authors reported a high efficacy of intratendinous PRP injection under ultrasound guidance. At 6 weeks and 32 months, the outcomes of clinical assessment and imaging tests were good or very good irrespective of the patient’s sex or the type of tendinopathy.

Fader et al. [78] reported the benefit of ultrasound-guided PRP injections in 18 patients at a mean age of 42.6 years with chronic proximal hamstring tendinopathy. In 10 patients (55.6%), an 80% reduction in VAS scores was achieved.

Wetzel et al. (118) [79] studied 17 patients with proximal hamstring injuries and compared the outcomes of PRP injections (12 patients) with nonsteroidal anti-inflammatory treatment and physiotherapy (5 patients). Patients treated with PRP injections showed a significant reduction in pain as measured by VAS and Nirschl Phase Rating Scale scores, in comparison with patients treated with a conservative approach.

Some studies assessed also the efficacy of ultrasound-guided percutaneous interventions for the hip. In a laboratory study of cadaveric specimens, Boetthcher et al. [80] assessed the feasibility of ultrasound-guided selective adductor longus release with a cutting wire as an alternative to surgical treatment. Of the 10 adductor longus tendons, 8 were completely transected, while in the remaining 2 cases, >99% of the tendon was transected. No injuries to adjacent structures were reported.

Bekgas et al. [81] conducted a prospective controlled randomized comparative study of 24 patients with greater trochanteric pain syndrome (GTPS) and showed that a single ultrasound-guided injection of 4 mL PRP was associated with better and longer-lasting clinical outcomes as compared with an ultrasound-guided injection of 4 mL methylprednisolone (40 mg/mL).

Ladurner et al. [82] conducted a systematic review of 27 studies, including more than 1100 patients. The use of PRP injections in grades 1 and 2 tendinopathy was shown to provide better outcomes than corticosteroid injections. The authors reported that 10% to 40% of patients with gluteal tendinopathy fail conservative treatment and require surgery.

Fitzpatrick et al. [83] compared the effectiveness of a single PRP injection and a single corticosteroid injection in a group of 80 patients at a mean age of 60 years and with a male-to-female ratio of 9:1. At 12 weeks, a significant improvement in HHS scores was shown in patients treated with PRP injections.

McInnis et al. [71] reviewed the current evidence for the use of orthobiologics in three common hip region conditions, including HOA, gluteal tendinopathy, and proximal hamstring tendinopathy. They concluded that the use of orthobiologics for hip conditions remains challenging, and the available evidence is limited and concerns only PRP injections.

Scientific reports on bio-orthopedic treatment note an average improvement in the local condition over a period of 12 months with an average of three PRP injections [72].

The above-mentioned controlled trials and meta-analyses have explored the role of orthobiologics, including PRP and BM-MSCs, in the treatment of HOA. While PRP injections have shown short- to mid-term benefits in pain and function, their effects tend to wane over time, and outcomes remain inconsistent across studies. Similarly, BM-MSC therapy demonstrates promising but preliminary efficacy, with methodological variability and limited long-term data. In contrast, our study introduces a standardized ultrasound-guided NSBT protocol, which resulted in more pronounced and sustained clinical improvements, suggesting that treatment precision and tissue targeting may play a key role in enhancing the therapeutic effect of orthobiologics. The present study demonstrates the potential of NSBT as a minimally invasive alternative to THA. However, future research should focus on elucidating the precise molecular mechanisms underlying its long-term effects. Advanced proteomic and transcriptomic analyses could provide deeper insights into the differential gene expression patterns induced by NSBT. Additionally, further investigation into the modulation of inflammatory and catabolic pathways in osteoarthritic joints will be essential to refine and optimize NSBT protocols for regenerative treatment.

The lack of a sham or placebo control is a limitation, particularly given the potential placebo effect of injection-based therapies. However, the study was designed to ensure that all participants received active treatment. Additionally, an imbalance in baseline OA grading was observed between the study groups, with a higher proportion of grade IV cases in the control group. Although participants were randomized, this discrepancy may have influenced outcome measures.

## 5. Conclusions

Based on the treatment outcomes for patients with OA of the hip joint, the NSBT method combined with the administration of RP-hCM has proven to be an effective and safe approach. It significantly reduces the need for surgical intervention through THA in patients with SHOA. This treatment does not necessitate hospitalization, anesthesia, or surgery, thereby minimizing all associated risk factors—both local and systemic—related to operative THA.

## Figures and Tables

**Table 1:** Baseline characteristics of the study and control groups.

	Study Group (n = 19)	Control Group (n = 19)
Age (years), mean (SD)	66.4 (14.2)	65.8 (16.7)
Sex, n (%)		
Men	10 (52.6)	8 (42.1)
Women	9 (47.4)	11(57.9)
Body mass index (kg/m^2^) (SD)	24.1 (2.2)	27.5 (4.1)
Grade, n (%)		
I	0	0
II	3 (15.8)	1 (5.3)
III	12 (63.2)	9 (47.35)
IV	4 (21.0)	9 (47.35)
SD—standard deviation		

**Table 2:** Clinical outcomes at baseline and at 12 months after treatment in the treatment and control groups.

		Study Group (N = 19)		Control Group (N = 19)		*p*
Variable		Mean	95% CI	Mean	95% CI	
VAS	At baseline	7.8	6.9–8.6	8.8	8.3–9.3	0.09
	After treatment	0.2	0.0–0.4	7.3	6.3–8.2	<0.001
	Change	−7.6	−8.5–−6.6	−1.5	−2.2–−0.8	<0.001
	*p* for change	<0.001		0.004		
WOMAC	At baseline	76.2	71.1–81.3	88.7	85.1–92.3	0.0002
	After treatment	10.5	5.1–15.9	78.3	73.2–83.4	<0.001
	Change	−65.7	−73.3–−58.1	−10.4	−19.9–−6.9	<0.001
	*p* for change	<0.001		<0.001		
HSS	At baseline	56.4	51.3–61.5	48.7	42.4–55.0	0.052
	After treatment	93.0	90.5–95.5	53.4	47.5–59.3	<0.0001
	Change	36.6	30.9–42.2	4.7	2.5–6.8	<0.0001
	*p* for change	<0.001		0.004		
RoM flexion	At baseline	94.0	91.6–96.3	90.8	87.8–93.8	0.09
	After treatment	139.2	136.6–141.8	92.63	90.2–95.1	<0.001
	Change	45.3	42.8–47.7	1.84	0.6–3.0	<0.001
	*p* for change	<0.001		0.24		
RoM ER	At baseline	9.7	8.4–10.6	8.7	7.6–9.7	0.29
	After treatment	28.4	27.0–29.8	11.6	10.4–12.7	<0.001
	Change	18.7	17.7–20.2	2.9	1.7–4.1	<0.001
	*p* for change	<0.001		0.012		
RoM IR	At baseline	21.6	18.7–24.5	21.7	19.0–24.4	0.95
	After treatment	61.3	57.3–65.3	26.6	24.5–28.7	<0.001
	Change	39.7	36.2–43.3	4.9	1.4–8.4	<0.001
	*p* for change	<0.001		0.048		

ER—external rotation; HHS—Harris Hip Score; IR—internal rotation; RoM—range of motion; WOMAC—Western Ontario and McMaster Universities Arthritis Index; VAS—Visual Analog Scale.

## Data Availability

The datasets generated and/or analyzed during the current study are available from the corresponding author on reasonable request.

## Supplementary Materials

The following supporting information can be downloaded at: https://www.mdpi.com/article/10.3390/biomedicines13040987/s1, Table S1: Sensitivity analysis after exclusion patients with grade IV OA.
