# Supplementary material for: Nanosurgery and Bioengineered Regenerative Protocols for the Treatment of Hip Osteoarthritis: A Double-Blind Randomized Controlled Trial as an Alternative to Surgical Hip Replacement
Source: Biomedicines. 2025 Apr 17;13(4):987. doi: 10.3390/biomedicines13040987 (PMC12024760; doi:10.3390/biomedicines13040987)
Supplement: Supplementary file 1 [file biomedicines-13-00987-s001.zip › biomedicines-3527033-supplementary.pdf]

Table S1. Sensitivity analysis after exclusion patients with grade IV OA.

		Study group (N=15)		Control group (N=10)		p
Variable		Mean	95% CI	Mean	95% CI	
VAS	At baseline	7.3	6.4-8.2	8.7	8.2-9.2	0.02
	After treatment	0.2	0.0-0.4	6.0	4.8-7.2	<0.001
	Change	7.1	6.0-8.2	2.7	1.7-3.7	<0.001
	p for change	<0.001		<0.001		
WOMAC	At baseline	74.8	69.1-80.7	85.2	81.0-89.4	0.01
	After treatment	9.1	3.7-14.5	73.0	67.2-78.8	<0.001
	Change	65.8	-58.1- -73.5	-12.2	-12.2 (-8.8- -15.6)	<0.001
	p for change	<0.001		<0.001		
HSS	At baseline	59.0	53.7-64.3	49.4	44.3-54.5	0.03
	After treatment	92.9	89.7-96.1	57.6	52.8-62.4	<0.001
	Change	33.9	27.7-40.1	8.2	11.8-4.6	<0.001
	p for change	<0.001		<0.001		
RoM flexion	At baseline	94.7	92.0-97.4	94.5	90.6-98.4	0.99
	After treatment	140.7	138.6-142.8	95.0	91.2-98.8	<0.001
	Change	46.0	43.0-49.0	0.5	0.6-1.6	<0.001
	p for change	<0.001		<0.001		
RoM ER	At baseline	9.3	7.9-10.7	9.0	7.5-10.5	0.99
	After treatment	28.7	27.1-30.3	12.0	10.2-13.8	<0.001
	Change	19.3	17.9-20.7	3.0	1.2-4.8	<0.001
	p for change	<0.001		<0.001		
RoM IR	At baseline	24.0	22.1-25.9	23.0	21.2-24.8	0.99
	After treatment	62.7	58.7-66.7	27.5	23.3-31.7	<0.001
	Change	38.7	34.7-42.7	4.5	0.6-8.4	<0.001
	p for change	<0.001		<0.001		
